# Effects of a Closed Space Environment on Gene Expression in Hair Follicles of Astronauts in the International Space Station

**DOI:** 10.1371/journal.pone.0150801

**Published:** 2016-03-30

**Authors:** Masahiro Terada, Masaya Seki, Rika Takahashi, Shin Yamada, Akira Higashibata, Hideyuki J. Majima, Masamichi Sudoh, Chiaki Mukai, Noriaki Ishioka

**Affiliations:** 1 Divison of Aerospace Medicine, The Jikei University School of Medicine, Minato-ku, Tokyo, Japan; 2 Japan Aerospace Exploration Agency, Tsukuba City, Ibaraki, Japan; 3 Space Biosciences Division, NASA Ames Research Center, Moffett Field, California, United States of America; 4 Advanced Engineering Services Co., Ltd., Takezono, Tsukuba City, Ibaraki, Japan; 5 Graduate School of Medical and Dental Sciences, Kagoshima University, Kagoshima City, Kagoshima, Japan; 6 Institute of Space and Astronautical Science, Sagamihara, Kanagawa, Japan; 7 Department of Space and Astronautical Science, School of Physical Sciences, SOKENDAI (The Graduate University for Advanced Studies), Sagamihara, Kanagawa, Japan; Texas Tech University, UNITED STATES

## Abstract

Adaptation to the space environment can sometimes pose physiological problems to International Space Station (ISS) astronauts after their return to earth. Therefore, it is important to develop healthcare technologies for astronauts. In this study, we examined the feasibility of using hair follicles, a readily obtained sample, to assess gene expression changes in response to spaceflight adaptation. In order to investigate the gene expression changes in human hair follicles during spaceflight, hair follicles of 10 astronauts were analyzed by microarray and real time qPCR analyses. We found that spaceflight alters human hair follicle gene expression. The degree of changes in gene expression was found to vary among individuals. In some astronauts, genes related to hair growth such as FGF18, ANGPTL7 and COMP were upregulated during flight, suggesting that spaceflight inhibits cell proliferation in hair follicles.

## Introduction

Long-term stay (~6 months per mission) in the International Space Station (ISS) has become a regular occurrence in recent times and will be necessary for explorations to Mars. When humans remain in space for an extended period, their bodies become adapted to the space environment. During long-term flight, physiological changes seen in astronauts include muscle atrophy and bone calcium loss. Furthermore, responses to radiation and adverse psychological effects are also important concerns [1]. Greenleaf et al. reported that astronauts lose their aerobic power and muscle strength and experience deterioration in mood and psychological state [2]. These adaptations sometimes pose physiological problems on return to earth. Therefore, the development of astronaut-specific healthcare technologies is very important. To achieve this purpose, it is necessary to have a better understanding of the physiological changes induced by the space environment such as microgravity and space radiation. In particular, understanding changes in protein and gene expression is important for developing countermeasures against the adverse effects experienced by astronauts who are in space for long duration missions. Since December 2009, the Japan Aerospace Exploration Agency (JAXA) has been conducting a clinical investigation to study the effects of long-term spaceflight on gene expression and mineral metabolism by analyzing hair samples from ISS crewmembers that have been in space (experiment nicknamed “HAIR”). Ten astronauts from the ISS crew participated in this HAIR experiment. Hair is one of the most suitable biological specimens for analysis, because it can generally be obtained by non-invasive and relatively easy procedures. We believe that the results of hair analysis will reflect the physiological conditions experienced during spaceflights. Therefore, the purpose of this experiment is to obtain information regarding the response of the hair follicle to the spaceflight environment. This information may contribute to the development of diagnostic methods to evaluate the health condition of astronauts during space missions.

Hair matrix cells actively divide in a hair follicle [3, 4] and are known to sensitively reflect the host’s physical conditions [5, 6, 7]. Akashi et al. reported that the circadian phase of clock gene expression in hair follicle cells corresponds to that of individual behavioral rhythms, and is therefore effective for evaluating the properties of human peripheral circadian clock [8]. In addition, the hair shaft has been shown to respond to the metabolic changes occurring in the organism in response to changing environments [9, 10]. For example, high levels of toxic metals such as mercury, cadmium, arsenic, and lead have been observed in the hair of individuals exposed to toxic metal pollution [11]. Hair mineral analysis has also been widely used for forensic science, assessment of environmental exposure [12, 13, 14, 15], evaluation of nutritional status, and disease diagnosis [16, 17]. It was previously reported that 14 days of hindlimb suspension (a simulated microgravity model of skeletal muscle) led to changes in the levels of 26 minerals in rat fur [18], supporting the idea that hair samples can be an informative tool for examining the effects of spaceflight on human beings, especially because no special complex hardware or handling is required for sample collection.

As part of the “HAIR” experiment, both hair follicles and shafts collected from the ISS crewmembers were subjected to analysis. The expression of immunoglobulin heavy-chain mRNA in the amphibian *Pleurodeles waltl* changes during spaceflight [19]. Spaceflight has also been shown to alter gene expression in rat and mouse skeletal muscles [20, 21, 22]. In addition, studies have suggested that spaceflight affects the organization of microtubules and mitochondria, thereby causing increased apoptosis [23]. Recently, analysis of skin from space-flown mice showed that the number of hair follicles in anagen decreased in space, and that prolonged exposure to space conditions might induce skin atrophy and dysregulate the hair follicle cycle [24]. In this study, we focused on the effects exerted by the space environment on hair follicle genes. Therefore, nucleic acids (RNA and mitochondrial DNA) were extracted from the hair follicles and subjected to gene expression analysis. The extracted total RNA was analyzed by microarray, and the effects of spaceflight on the expression levels of hair follicle genes and marker genes of human epithelial hair follicle stem cells were further examined.

## Materials and Methods

### Hair sample preparation

**Fig 1:**
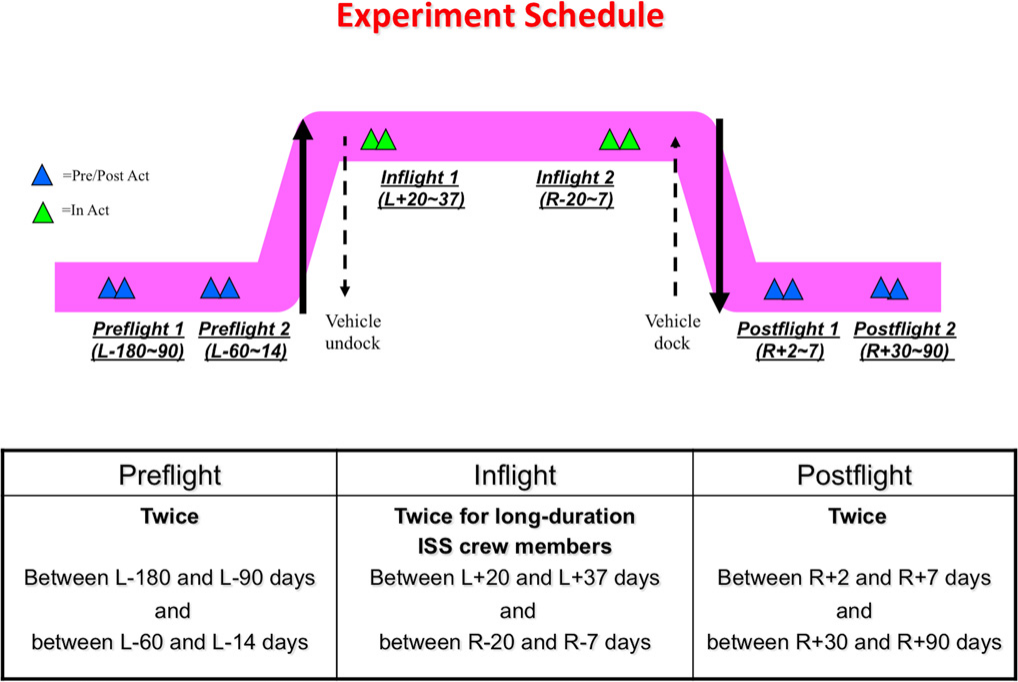
Experiment schedule. At each sampling point, two astronauts were paired and each astronaut took hair samples from the other astronaut by turns (sample number indicated in the text). L: Launch, R: Return.

Ten astronauts (male = 8, female = 2) at the International Space Station (ISS) participated in the study. They were at ISS on a 6-month-long mission. In each mission, five strands of hair were sampled six times from each astronaut within the period of July 2009 to February 2013. The sampling timings were as follows: 6 sampling time points (Fig 1); first preflight (Launch (L) -180 to -90 days: 6 to 3 months before launch), second preflight (L-60 to -14: 2 months to 2 weeks before launch), first inflight (L+20 to 37: 20 to 37 days after launch), second inflight (Return (R) -20 to -7: 20 to 7 days before return), first postflight (R+2 to 7: 2 to 7 days after return), and second postflight (R+30 to 90: from 1 to 3 months after return). The sampling days differed for each astronaut because of differing schedules. In each mission, two astronauts were paired and individual hair samples were collected. At one time point, five strands of hairs were grasped as close as possible to the scalp and pulled out using tweezers in the direction of hair growth without damaging the hair follicles. The samples were stored at -80°C until analysis at the preflight and postflight sampling points. In space, the samples were stored at Minus Eighty Degree Laboratory Freezer for ISS (MELFI) as soon as possible until return.

This study was approved by the Committee on Human Care and Use at the NASA and JAXA Ethical Review Board and the Human Research Multilateral Review Board (HRMRB). All participants provided written informed consent.

### RNA extraction

Hair follicles (approximately 2–3 mm) were used as the source for mRNA extraction; the follicles were cut into ~15 fragments (0.1–0.2 mm each) by using a microsurgical knife under a stereoscopic microscope. Collected fragments were immersed in 800 μl of ISOGEN Reagent (Nippon Gene, Toyama, Japan) in tubes and stirred (15 s × 2 times) by using the sonication device Bioruptor UCD-250 (Cosmo Bio, Tokyo, Japan). Next, RNA was purified from hair lysates using ISOGEN Kit as described previously [25]. Briefly, the tubes were maintained at room temperature for 5 min, followed by the addition of 200 μl of chloroform. Subsequent processes of RNA purification were performed according to manufacturer’s instructions. After isolation, RNA pellets were washed with 70% ethanol, air dried, and resuspended in 10 μl of RNA-free water (Gibco-BRL, Gaithersburg, MD). Total RNA was quantified at 260 nm using NanoDrop ND-1000 spectrophotometer (NanoDrop Technologies Inc., Wilmington, DE). RNA quality was determined using Agilent Bioanalyzer 2100 (Agilent Technologies, Palo Alto, CA). The 28S:18S rRNA ratio and the RNA integrity number (RIN) were calculated using 2100 Expert software and RIN Beta Version software (Agilent Technologies), respectively. RIN was calculated by using the classification of total RNA, based on a numbering system from 1 (most degraded) to 10 (most intact) [26, 27]. RNA yields and RIN number are described in Table 1. The RIN numbers in some samples were low probably due to higher extent of RNA degradation or due to low detection of 28S:18S rRNA ratio in the low concentration samples.

**Table 1:** RNA yield and RIN number.

Sample		Conc. (ng/uL)	RIN	Sample		Conc. (ng/uL)	RIN
**1**	**Pre 1**	91.6	5.9	**6**	**Pre 1**	33.71	8.9
** **	**Pre 2**	44.6	6.8	** **	**Pre 2**	21.89	8.7
** **	**Infligt 1**	180.2	4.7	** **	**Infligt 1**	33.57	4.8
** **	**Inflight 2**	208.9	4.6	** **	**Inflight 2**	108.38	7.6
** **	**Post 1**	63.4	5.6	** **	**Post 1**	191.99	7.0
** **	**Post 2**	36.0	6.3	** **	**Post 2**	209.07	5.4
**2**	**Pre 1**	67.36	5.7	**7**	**Pre 1**	6.63	1.0
** **	**Pre 2**	115.11	5.7	** **	**Pre 2**	35.26	7.4
** **	**Infligt 1**	143.6	6.2	** **	**Infligt 1**	28.67	9.3
** **	**Inflight 2**	148.42	4.9	** **	**Inflight 2**	51	8.0
** **	**Post 1**	88.69	7.3	** **	**Post 1**	44.18	7.7
** **	**Post 2**	91.46	2.4	** **	**Post 2**	31.08	6.3
**3**	**Pre 1**	177.21	7.1	**8**	**Pre 1**	37.7	7.4
** **	**Pre 2**	121.23	6.2	** **	**Pre 2**	23.79	8.7
** **	**Infligt 1**	165.29	6.8	** **	**Infligt 1**	110.51	7.9
** **	**Inflight 2**	204.59	5.7	** **	**Inflight 2**	26.36	1.1
** **	**Post 1**	179.59	5.5	** **	**Post 1**	70.15	7.1
** **	**Post 2**	49.19	8.1	** **	**Post 2**	33.61	5.6
**4**	**Pre 1**	49.03	5.4	**9**	**Pre 1**	43.73	1.0
** **	**Pre 2**	44.6	2.5	** **	**Pre 2**	46.63	7.0
** **	**Infligt 1**	48.49	5.9	** **	**Infligt 1**	50.77	8.4
** **	**Inflight 2**	49.14	2.4	** **	**Inflight 2**	44.35	8.1
** **	**Post 1**	34.38	5.3	** **	**Post 1**	40.72	8.4
** **	**Post 2**	55.08	5.7	** **	**Post 2**	40.42	7.3
**5**	**Pre 1**	25.72	1.9	**10**	**Pre 1**	89.92	8.3
** **	**Pre 2**	30.19	2.1	** **	**Pre 2**	377.37	7.7
** **	**Infligt 1**	36.64	2.5	** **	**Infligt 1**	90.48	6.9
** **	**Inflight 2**	198.65	6.4	** **	**Inflight 2**	148.12	6.1
** **	**Post 1**	34.99	N/A	** **	**Post 1**	306.67	6
** **	**Post 2**	47.81	N/A	** **	**Post 2**	41.91	6.3

### RNA amplification

Because of the small amount of RNA extracted from hair samples, a double RNA amplification step was incorporated prior to microarray hybridization. Total RNA was amplified using the Ambion MessageAmp aRNA Kit as described previously [28]. Briefly, first- and second-strand cDNA were synthesized. Unlabeled amplified (aRNA) was generated by *in vitro* transcription with non-biotinylated NTPs. For probe preparation, amplified aRNA was reverse-transcribed with second-round primers. The second-strand cDNA was synthesized with T7 oligo (dT) primer and purified. Biotin-labeled cRNA was generated by *in vitro* transcription and purified with the RNeasy Kit (Qiagen, Venlo, Netherlands).

### Generation and mining of microarray data

Amplified RNA was processed and hybridized to the Whole Human Genome (8 × 60K) Oligo Microarray (Agilent Technologies), according to manufacturer’s protocol. Slide scanning was performed using the Agilent DNA Microarray Scanner (Agilent Technologies) by DNA Chip Research Inc. (Yokohama, Japan). Expression profiles were collected in triplicate at each time point, and scanning data were normalized with Agilent’s Feature Extraction software (Agilent Technologies). Data preprocessing and analysis were performed using the GeneSpring software 11.0.1 (Agilent Technologies). Preprocessing was performed according to manufacturer’s recommendations and MicroArray Quality Control project reports [29]. Briefly, a decision matrix determines whether each transcript is reliably detected (i.e., present), marginally detected (i.e., marginal), or not detected (i.e., absent), and calculates the signal intensity. Normalization was performed to the 75^th^ percentile of each array, and each gene to the median, with GeneSpring’s normalization option. Hierarchical cluster analysis was performed using principal component analysis (PCA), rank correlation of log ratios, and condition tree clustering options of GeneSpring. The probability was 0.1 and was adjusted by the false-discovery rate for correction of multiple tests. All raw fluorescence intensity data and microarray image files were deposited within the public repository for microarray-based gene expression data, the “Gene Expression Omnibus” (GEO) (http://www.ncbi.nlm.nih.gov/geo/), complying with the minimum information requirement for microarray experiments. The GEO accession number for the current experiment is GSE74708.

### Quantitative PCR (qPCR)

Synthesis of cDNA (1 μg) was carried out using the PrimeScript RT reagent Kit (TaKaRa Bio, Shiga, Japan) following the manufacturer’s instructions. The primers were designed using Primer Express 3.0 (Applied Biosystems, Foster, CA). Primer pair sequences are described in Table 2. The qPCR experiment was performed with SYBR Premix Ex Taq (TaKaRa Bio) using the 7500 Real-Time PCR system (Applied Biosystems). The changes in gene expression were analyzed by the relative quantitative method in a given sample relative to the control sample, and specific mRNA transcript levels were expressed as fold difference. The results were normalized to the corresponding intensity of 18S rRNA as an internal control. Each primer/cDNA set was checked in duplicate from two independent samples.

**Table 2:** Primer pairs used in the study.

Gene	Primer Type	Sequence
FGF18	Forward Primer	CGCCCAGCGATGTATTCAG
	Reverse Primer	ATGCGGAAGTCCACGTTCTC
ANGPTL7	Forward Primer	CGCAAAGGTGGCTACTGGTACA
	Reverse Primer	TAGGTAGATCCATGCCAGCCATA
CDK1	Forward Primer	GGGCACTCCCAATAATGAAGTG
	Reverse Primer	TCGTTTGGCTGGATCATAGATTAAC
COMP	Forward Primer	GACAGTGATGGCGATGGTATAGG
	Reverse Primer	GTCACAAGCATCTCCCACAAAGT

### Statistical analyses

Values are expressed as mean ± SEM. Significant differences among groups were determined by analysis of variance, followed by Scheffé’s post-hoc test. Differences were considered significant at a confidence level of 0.05.

## Results

### Gene expression affinity analysis

**Fig 2:**
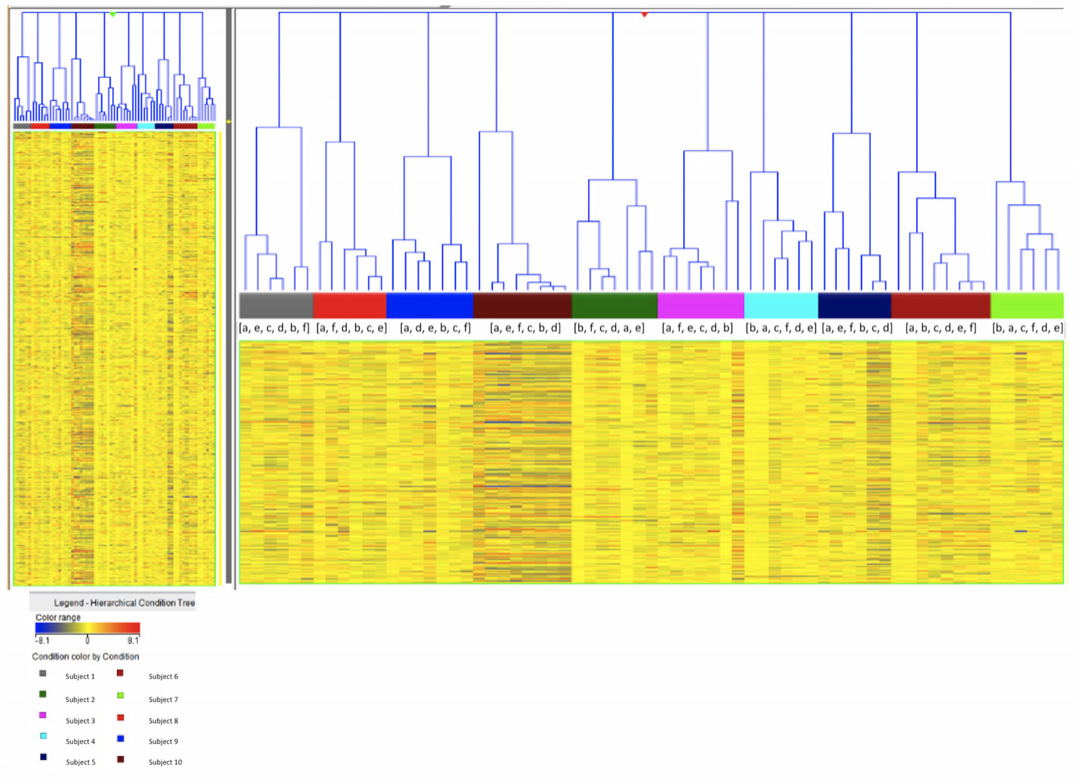
Hierarchical cluster analysis by condition tree clustering. Each individual color corresponds to a data from a single astronaut. Data from 10 a total of astronauts are presented. Letters indicate sampling time point: (a) Preflight 1, (b) Preflight 2, (c) Inflight 1, (d) Inflight 2, (e) Postflight 1, (f) Postflight 2.

Microarray analysis was performed for total RNA extracted from hair follicles. To evaluate the homogeneity among multiple genes in all samples, hierarchical analysis was performed using condition tree clustering (Fig 2). In this figure, each lattice bar indicates each sampling time. Each color band below these lattice bars indicates the data from each astronaut. These results indicate clear resolution between the subjects. The values obtained at six different sampling times for each astronaut lie next to each other and divide per each astronaut occur within a narrow range (Fig 2). The differences between individuals appear to be larger than those observed at six different sampling points from the same subject, irrespective of length of stay in space.

### Hair follicle-related gene expression patterns

**Fig 3:**
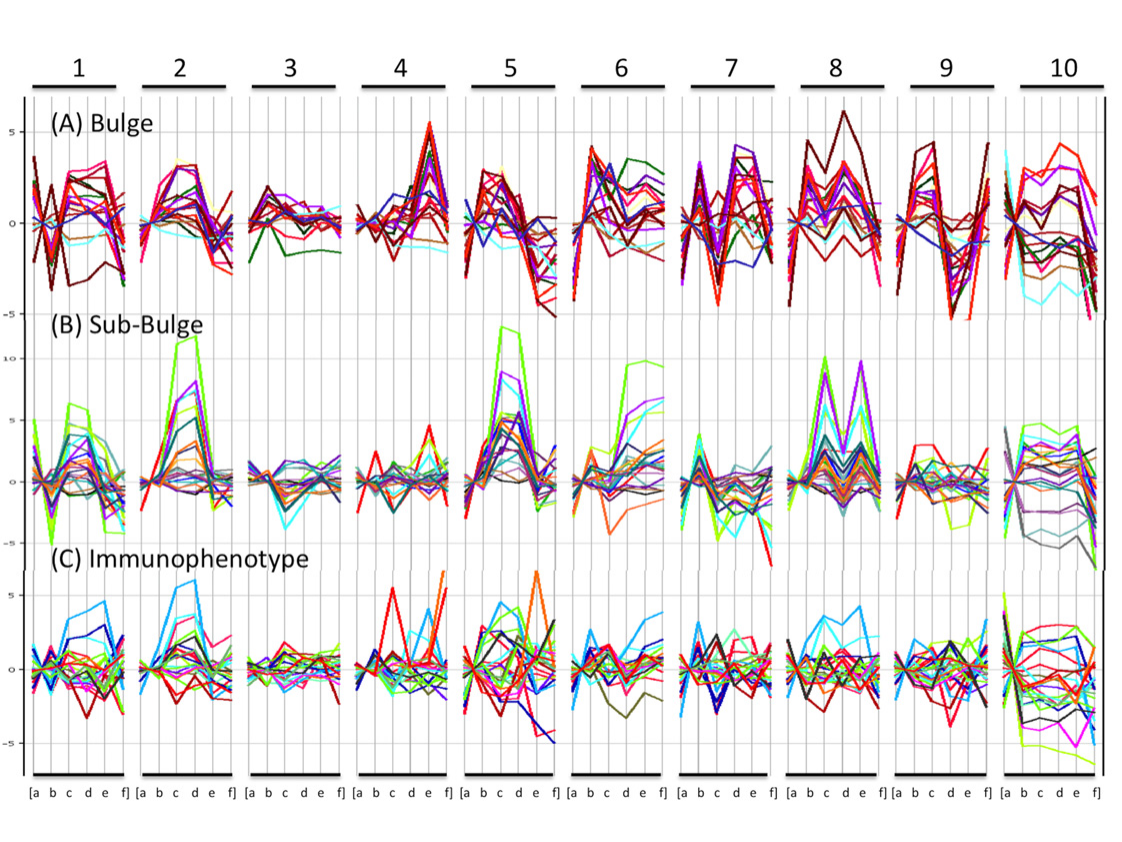
Expression of hair follicle genes in 10 astronauts. Gene expression (microarray) data from 10 astronauts. The number headings indicate subject ID number. Small letters indicate sampling time point: (a) Preflight 1, (b) Preflight 2, (c) Inflight 1, (d) Inflight 2, (e) Postflight 1, (f) Postflight 2. (A) Genes associated with hair bulge. (B) Genes associated with hair sub-bulge. (C) Marker genes of human epithelial hair follicle stem cells (immunophenotype genes).

To confirm the integrity of RNA extracted from hair, expression levels of hair-specific genes in these samples were analyzed. The average value of first preflight and second preflight sampling points was used as baseline. Hair follicle specific genes were chosen based on the studies by Ohyama et al. [30] and Kloepper et al. [31]. The changes in the expression of 14 reported genes (Table 3A) in the bulge region of hair follicles were observed in the microarray data at all sampling points (Fig 3A). Similarly, the expression patterns of 22 genes in the hair sub-bulge region (Table 3B) were confirmed (Fig 3B). In addition, the expression patterns of 16 marker genes of human epithelial hair follicle stem cells (referred to as “immunophenotype genes” by Kloepper et al.) [31] (Table 3C) were detected (Fig 3C).

**Table 3:** List of hair follicle-associated genes.

**(A) Genes associated with hair bulge.**	
**GeneSymbol**	**Description**
SERPINF1	Homo sapiens serpin peptidase inhibitor, clade F (alpha-2 antiplasmin, pigment epithelium derived factor), member 1 (SERPINF1), mRNA [NM_002615]
FST	Homo sapiens follistatin (FST), transcript variant FST344, mRNA [NM_013409]
DCN	Homo sapiens decorin (DCN), transcript variant E, mRNA [NM_133507]
DIO2	Homo sapiens deiodinase, iodothyronine, type II (DIO2), transcript variant 1, mRNA [NM_013989]
ANGPTL2	Homo sapiens angiopoietin-like 2 (ANGPTL2), mRNA [NM_012098]
PHLDA1	Homo sapiens pleckstrin homology-like domain, family A, member 1 (PHLDA1), mRNA [NM_007350]
DCT	Homo sapiens dopachrome tautomerase (dopachrome delta-isomerase, tyrosine-related protein 2) (DCT), transcript variant 1, mRNA [NM_001922]
FZD1	Homo sapiens frizzled family receptor 1 (FZD1), mRNA [NM_003505]
KRT15	Homo sapiens keratin 15 (KRT15), mRNA [NM_002275]
DPYSL2	Homo sapiens dihydropyrimidinase-like 2 (DPYSL2), transcript variant 2, mRNA [NM_001386]
DPYSL3	Homo sapiens dihydropyrimidinase-like 3 (DPYSL3), transcript variant 2, mRNA [NM_001387]
DKK3	Homo sapiens dickkopf homolog 3 (Xenopus laevis) (DKK3), transcript variant 1, mRNA [NM_015881]
WIF1	Homo sapiens WNT inhibitory factor 1 (WIF1), mRNA [NM_007191]
TGFB2	Homo sapiens transforming growth factor, beta 2 (TGFB2), transcript variant 2, mRNA [NM_003238]
**(B) Genes associated with hair sub-bulge.**	
**GeneSymbol**	**Description**
TIMP3	Homo sapiens TIMP metallopeptidase inhibitor 3 (TIMP3), mRNA [NM_000362]
COL11A1	Homo sapiens collagen, type XI, alpha 1 (COL11A1), transcript variant B, mRNA [NM_080629]
TYMS	Homo sapiens thymidylate synthetase (TYMS), mRNA [NM_001071]
ANGPTL7	Homo sapiens angiopoietin-like 7 (ANGPTL7), mRNA [NM_021146]
CDK1	Homo sapiens cyclin-dependent kinase 1 (CDK1), transcript variant 1, mRNA [NM_001786]
TOP2A	Homo sapiens topoisomerase (DNA) II alpha 170kDa (TOP2A), mRNA [NM_001067]
PDGFC	Homo sapiens platelet derived growth factor C (PDGFC), transcript variant 1, mRNA [NM_016205]
SDC2	Homo sapiens syndecan 2 (SDC2), mRNA [NM_002998]
SLC7A1	Homo sapiens solute carrier family 7 (cationic amino acid transporter, y+ system), member 1 (SLC7A1), mRNA [NM_003045]
LAMB1	Homo sapiens laminin, beta 1 (LAMB1), mRNA [NM_002291]
VAV3	Homo sapiens vav 3 guanine nucleotide exchange factor (VAV3), transcript variant 1, mRNA [NM_006113]
COMP	Homo sapiens cartilage oligomeric matrix protein (COMP), mRNA [NM_000095]
PCDH8	Homo sapiens protocadherin 8 (PCDH8), transcript variant 1, mRNA [NM_002590]
KPNA2	Homo sapiens karyopherin alpha 2 (RAG cohort 1, importin alpha 1) (KPNA2), mRNA [NM_002266]
GPC4	Homo sapiens glypican 4 (GPC4), mRNA [NM_001448]
RRM2	Homo sapiens ribonucleotide reductase M2 (RRM2), transcript variant 2, mRNA [NM_001034]
PRC1	Homo sapiens protein regulator of cytokinesis 1 (PRC1), transcript variant 1, mRNA [NM_003981]
SLC1A4	Homo sapiens solute carrier family 1 (glutamate/neutral amino acid transporter), member 4 (SLC1A4), transcript variant 1, mRNA [NM_003038]
FEN1	Homo sapiens flap structure-specific endonuclease 1 (FEN1), mRNA [NM_004111]
SLC4A7	Homo sapiens solute carrier family 4, sodium bicarbonate cotransporter, member 7 (SLC4A7), mRNA [NM_003615]
MCAM	Homo sapiens melanoma cell adhesion molecule (MCAM), mRNA [NM_006500]
FGF18	Homo sapiens fibroblast growth factor 18 (FGF18), mRNA [NM_003862]
**(C) Marker genes for human epithelial hair follicle stem cells.**	
**GeneSymbol**	**Description**
CD200	Homo sapiens CD200 molecule (CD200), transcript variant 2, mRNA [NM_001004196]
ITGB1	Homo sapiens integrin, beta 1 (fibronectin receptor, beta polypeptide, antigen CD29 includes MDF2, MSK12) (ITGB1), transcript variant 1E, mRNA [NM_133376]
CD34	Homo sapiens CD34 molecule (CD34), transcript variant 2, mRNA [NM_001773]
GJA1	Homo sapiens gap junction protein, alpha 1, 43kDa (GJA1), mRNA [NM_000165]
LHX2	Homo sapiens LIM homeobox 2 (LHX2), mRNA [NM_004789]
ITGA6	Homo sapiens integrin, alpha 6 (ITGA6), transcript variant 2, mRNA [NM_000210]
KRT15	Homo sapiens keratin 15 (KRT15), mRNA [NM_002275]
LTBP1	Homo sapiens latent transforming growth factor beta binding protein 1 (LTBP1), transcript variant 1, mRNA [NM_206943]
NES	Homo sapiens nestin (NES), mRNA [NM_006617]
TNC	Homo sapiens tenascin C (TNC), mRNA [NM_002160]
FBN1	Homo sapiens fibrillin 1 (FBN1), mRNA [NM_000138]
FBN2	Homo sapiens fibrillin 2 (FBN2), mRNA [NM_001999]
FBN3	Homo sapiens fibrillin 3 (FBN3), mRNA [NM_032447]
FN1	Homo sapiens fibronectin 1 (FN1), transcript variant 7, mRNA [NM_054034]
NID1	Homo sapiens nidogen 1 (NID1), mRNA [NM_002508]
NID2	Homo sapiens nidogen 2 (osteonidogen) (NID2), mRNA [NM_007361]

Three of the 10 astronauts (subject number 1, 2, and 5) showed an increase in gene expression during spaceflight irrespective of the hair follicle portion (bulge or sub-bulge); marker genes of human epithelial hair follicle stem cells also showed a similar trend (Fig 3A–3C). In subject number 6, 7, 8, 9, and 10, some genes showed high levels of variation before flight or after return. Subject number 3 and 4 showed relatively stable gene expression patterns at all sampling points. These two astronauts were female, hence suggesting gender-based differences in hair follicle response to flight.

### Detection of genes responsive to the space environment

**Fig 4:**
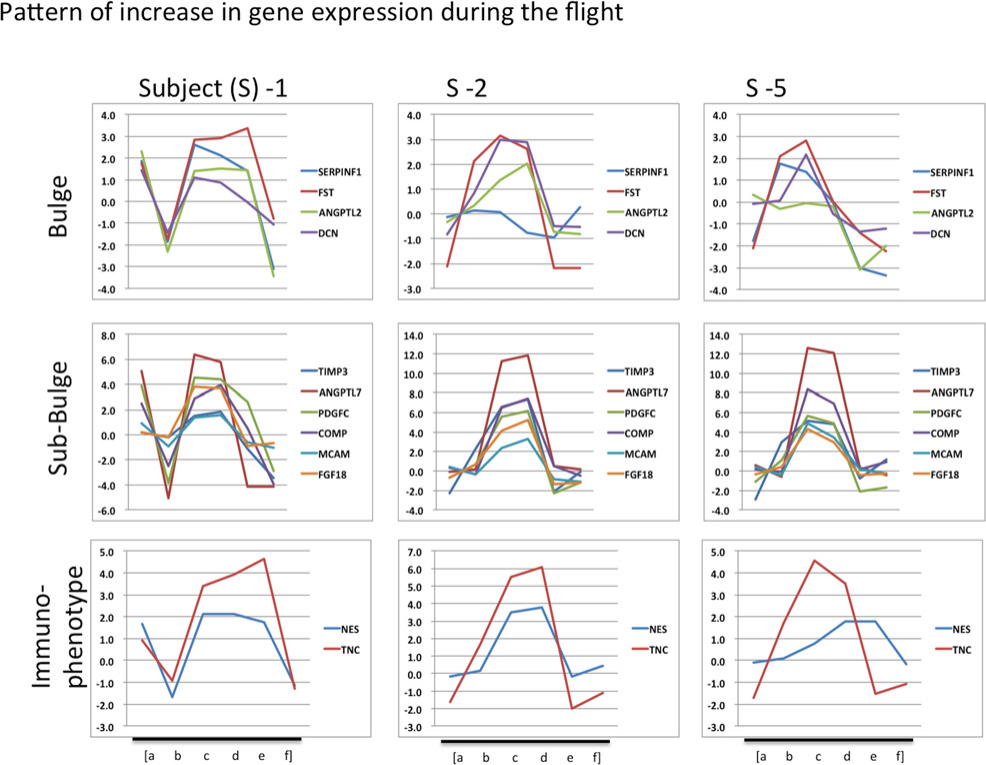
Expression of hair follicle genes in each astronaut. Several genes were selected from the data depicted in Fig 3. Data from astronauts with upregulated gene expression in space. Small letters indicated sampling timepoints: (a) Preflight 1, (b) Preflight 2, (c) Inflight 1, (d) Inflight 2, (e) Postflight 1, (f) Postflight 2.

**Fig 5:**
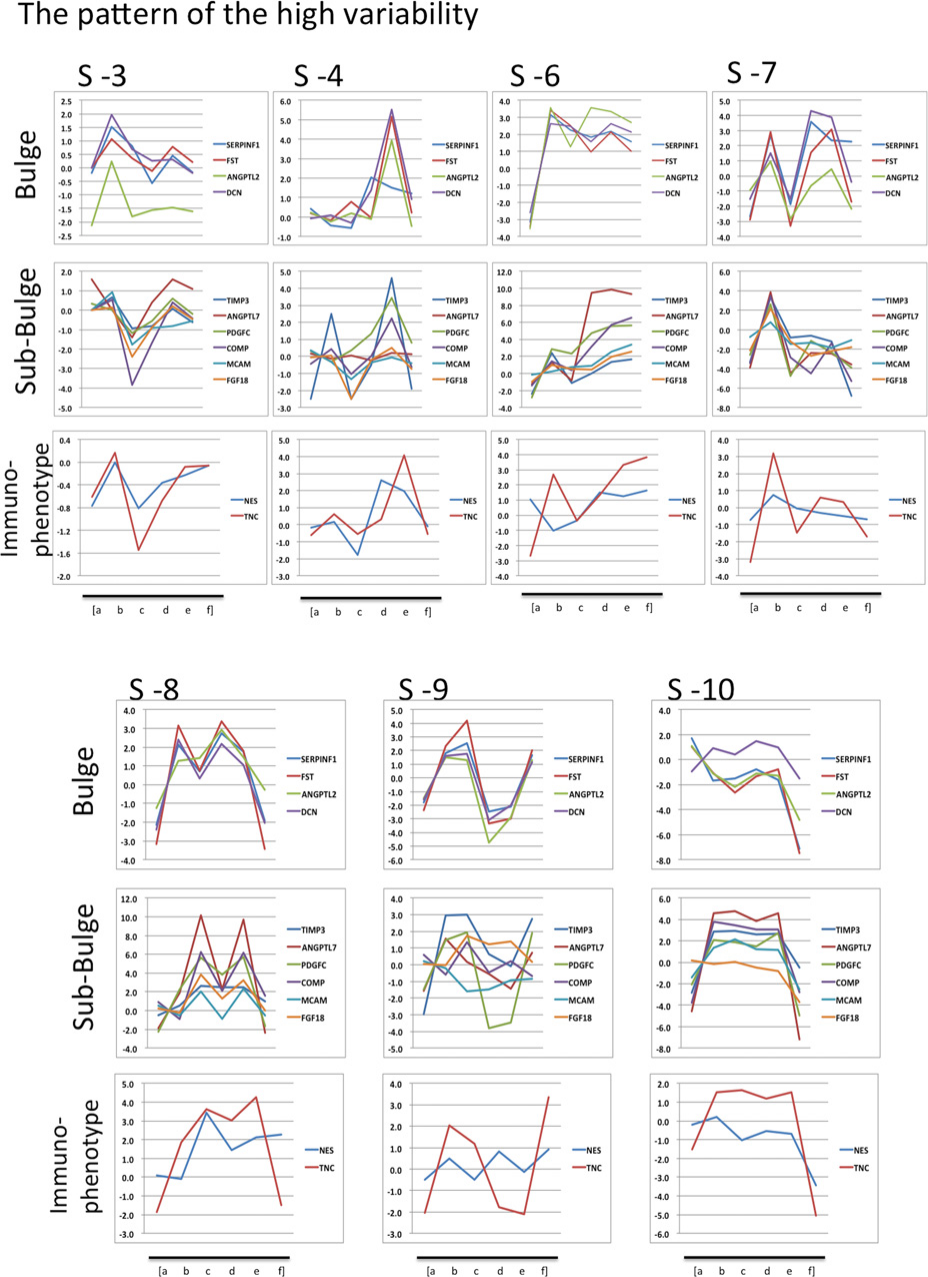
Expression of hair follicle genes in each astronaut. Several genes were selected from the data depicted in Fig 3. Data from astronauts with high variability in gene expression. Small letters indicated sampling timepoints: (a) Preflight 1, (b) Preflight 2, (c) Inflight 1, (d) Inflight 2, (e) Postflight 1, (f) Postflight 2.

A subset of genes from Table 3 was selected and corresponding microarray gene expression values were compared among subjects. In the bulge portion, four genes were selected: SERPINF1, FST, ANGPTL2, and DCN, and from the sub-bulge portion, six genes were chosen: TIMP3, ANGPIL7, PDGFC, COMP, MCAM, and FGF18. Two genes (NES and TNC) were selected from the marker genes of human epithelial hair follicle stem cells (immunophenotype genes). A comparison of these genes for each subject (subject number 1–10) is shown in Figs 4 and 5. Fig 4 shows the data from three astronauts, which shows an increase in gene expression during spaceflight. Fig 5 shows the data from other seven astronauts. Although the data from female astronauts (subject number 3 and 4) seemed relatively stable compared to the data from male astronauts (Fig 3), whose patterns showed high variability during all sampling points.

### Quantification of hair follicle gene expression

The expression of hair follicle genes was quantified by qPCR. In the “HAIR” experiment, the number of hair samples was limited to only 5 strands from each astronaut to minimize subject discomfort and scalp damage. Procedure optimization showed that 5 hair follicles was the minimum number that can generate sufficient RNA for analysis. The total RNA obtained from five hair strands was pooled into one tube at each sampling point. Each value in qPCR data was expressed as the average value of two technical replicates from the same tubes. Therefore, statistical evaluation was not undertaken.

**Fig 6:**
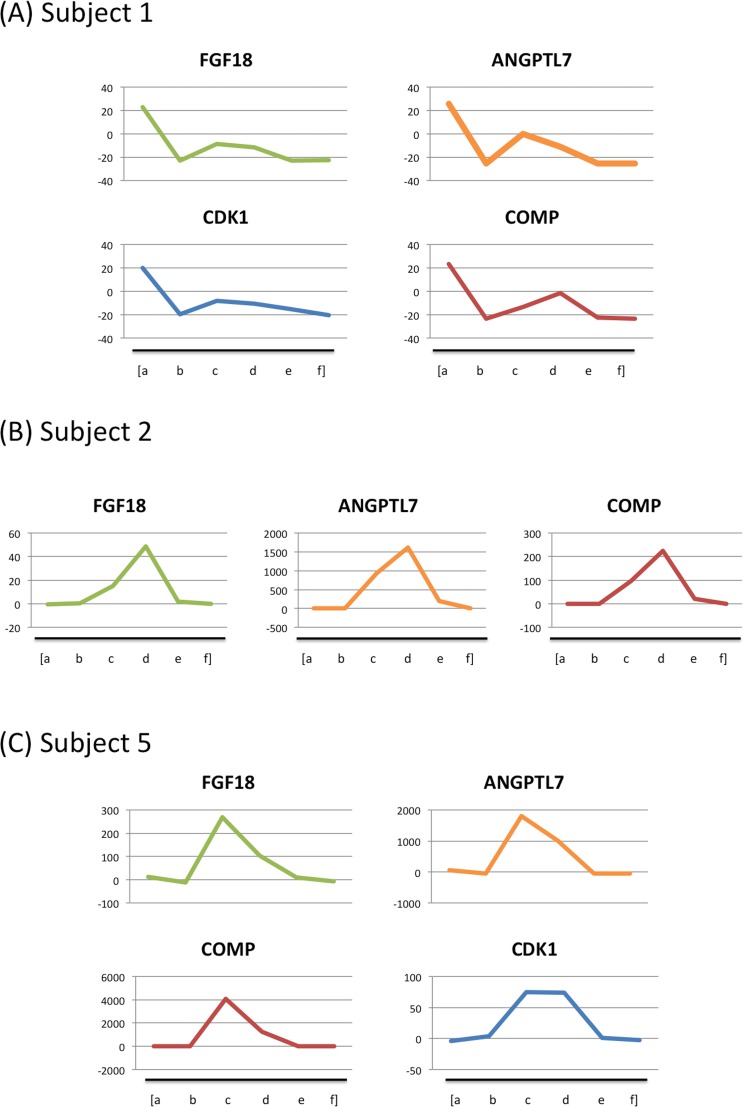
Quantitative PCR data from three astronauts. (A) Subject 1. (B) Subject 2. (C) Subject 5. Small letters indicate sampling points: (a) Preflight 1, (b) Preflight 2, (c) Inflight 1, (d) Inflight 2, (e) Postflight 1, (f) Postflight 2.

In Fig 6, the levels of some genes that were upregulated during the flight were determined from the qPCR data for three astronauts. Three subjects showed similar patterns of increase in gene expression during flight. These three astronauts also showed high variability in gene expression during flight (Figs 3 and 4). For subject 1 and 5 (Fig 6A and 6C), four genes were chosen: FGF18, ANGPTL7, CDK1, and COMP. For subject 2 (Fig 6B), only three genes were studied: FGF18, and ANGPTL7, and COMP. In these figures, because the samples taken before launch can be considered as the normal condition for astronauts, values from first (pre 1) and second preflight (pre 2) sampling points were averaged and used as the baseline data (Figs 3, 4 and 5). In subject number 1, all four genes showed an increasing pattern during flight (Fig 6A). CDK1 level was upregulated >70 times during the flight, compared with the baseline in subject number 5 (Fig 6C). For COMP gene, the level was >4,000 times higher during spaceflight in subject 5, although the level was >200 times higher in subject 2 (Fig 6B and 6C). For FGF18, the level of expression was >50 times and >250 times higher in subject 2 and 5, respectively. For ANGPTL7, the level of expression was >1,500 times higher, when compared to the baseline, in subject 2 and 5.

## Discussion

To date, there have been few studies on the effects of space environment on human hair. Recently, some studies using rodents were performed at ISS. Through the sample-sharing program, skins from space-flown mice were investigated by other groups [24, 32]. Their results suggested that space environment induced changes in gene expression in mouse skin. Our study is the first study that evaluates gene expression changes in astronauts at multiple timepoints of spaceflight. We found that FGF18 expression in the hair follicle changed during spaceflight. Hair follicle growth during anagen is strongly suppressed by the local delivery of FGF18 protein [33]. Epithelial FGF18 signaling and reduction of expression in the milieu of hair stem cells are crucial for the maintenance of resting and growth phases. On the other hand, Ohyama et al. reported that growth factors that are upregulated in the suprabulbar region of the outer root sheath (e.g., FGF18 and PDGFC) may enhance hair shaft growth and may have therapeutic efficacy in alopecia [30]. Leishman et al. reported that FGF18 controls the duration of the quiescent phase in the hair follicle [34]. FGF18 functions in the maintenance of the resting phase of the hair cycle; regulation of FGF18 expression is important to maintain the cycle of hair growth and arrest [34]. FGF18 expression is known to decrease in growing hair follicles [35]. The increase in FGF18 expression in several astronauts during flight potentially reflects a temporary arrest in the hair growth cycle. FGF18 expression appears to be very sensitive to whether an astronaut is in space or earth-bound; FGF18 easily recovered to baseline levels after returning to earth. These results suggest that the hair cycle is very responsive to environmental cues. Neutelings et al. investigated skin from space-flown mice [24] and reported that the number of hair follicles in anagen decreased in the space environment suggesting that prolonged exposure to space environment might induce skin atrophy and deregulate hair follicle cycle. Considering the function of FGF18 in hair, our results are consistent with their findings.

ANGPTL7 in the extracellular matrix of the trabecular meshwork plays an important role in the deposition and organization of the matrix of the outflow tissue [36,37]. In human hair, Ohyama et al. reported that ANGPTL7 was expressed as a growth factor in the hair follicle [30]. ANGPTL7 was reported to be downregulated in growing hair follicles [35]. In our experiments, ANGPTL7 expression was increased during spaceflight among some astronauts. The changes we detected in ANGPTL7 may also reflect the inhibition in growth of hair follicles in the space, similar to the results of FGF18 expression analysis.

COMP was found to be dependent on the hair cycle, expressed during telogen and early anagen and degraded during catagen [38]. From our results, COMP was upregulated during spaceflight in three astronauts. For two astronauts, CDK1 gene level was higher in space than preflight or postflight. It was reported that cyclin D1 showed a >3-fold increase in the mRNA from the telogen bulge [39]. Lindqvist et al. reported that knockdown of both Cdc25a and Cdc2b inhibits M-phase entry and reduced dephosphorylation and the activity of CDK1 in cultured cells [40]. Cdk1, which binds p21 with lower affinity than Cdk2, has an important role in abrogating the postmitotic checkpoint [41]. Changes in expression levels of these genes (COMP and CDK1) could contribute to disruption of the cell cycle in hair follicles we analyzed.

In addition to FGF18, ANGPTL7, COMP, and CDK1, several genes were compared by microarray analysis (Figs 4 and 5). Among these, the PDGF family is associated with organogenesis of the hair follicle [42]. Ohyama et al. reported that PDGFC was downregulated in the anagen phase of human hair bulge [30]. PDGF induced and maintained the anagen phase in mouse hair cycle [43, 44]. MCAM (CD146) has been recently identified as an integral member of the cell adhesion molecule (CAM) family [45, 46]. MCAM protein is frequently overexpressed on the surface of advanced and metastatic human melanoma cells; however, its expression is rare in benign nevi [46, 47, 48]. In hair follicle, this gene was preferentially expressed by non-bulge keratinocytes [30]. They also reported that MCAM was overrepresented in the sub-bulge outer root sheath. In our study, these two genes increased during spaceflight, again suggesting that the space environment alters hair growth.

Although protocadherin 8 (PCDH8) was listed in Table 3B, this gene has a role in morphogenesis and cell growth [49]. Some astronauts showed an increase in the expression of this gene in space (Fig 3). The loss of PCDH8 is known to promote oncogenesis in epithelial human cancers by disrupting cell-cell communication dedicated to tissue organization and repression of mitogenic signaling [49]. In human hair, Ohyama et al. suggested that PCDH8 was downregulated in the anagen phase of hair [30]. On the other hand, Mash et al. reported that PCDH8 was upregulated in human hippocampus in response to cocaine exposure [50]. PCDH8 might be affected by neurochemical adaptations to the space environment. Yamamoto et al. suggested that autonomic nervous functions and circadian rhythms were distributed in space [51]. Therefore, during spaceflight, several factors (such as mental factors) induce the increase in PCDH8 in hair, and the hair cycle might be arrested among some astronauts.

The expression of several genes increased during flight among some astronauts. A number of these responses to spaceflight have been detected for the first time. In addition, these responses appeared to be shared among a subset of astronauts tested.

Generally, human hair is affected by many factors, for example, diet or life style, psychological state, etc. These factors may have contributed to some variability in the gene expression results among subjects. Despite some degree of variation, common patterns in gene expression and general observations can be drawn. Firstly, gender appears to influence the response to spaceflight. The responses of female astronauts were slightly different from those of male astronauts. Although there are many differences such as hormone levels or functions between males and females, female astronauts appear to have a better response against the features of the space environment, as one example, FGF18 expression in females was more stable in space than in males.

Current limitations of this study include the modest sample size, due to the nature of spaceflight operations at the ISS. Also, we currently do not have access to metabolic data and are unable to correlate our gene expression data with physiological changes in these astronauts. In addition, the hair cycle is not controlled by single gene. Therefore, it is unclear whether our results reflect the inhibition of hair growth in space. However, these results are valuable to establish that spaceflight induces similar gene expression changes among different astronauts. Moreover, there is a possibility that the key factors will be identified in future studies for evaluating the conditions of various organs, by comparing our genetic data with that of other organs.

In conclusion, we found that spaceflight alters human hair follicle gene expression. Follow up studies are necessary to improve our understanding of the mechanisms underlying the physiological adaptations to spaceflight.
