# Correction: Effects of a Closed Space Environment on Gene Expression in Hair Follicles of Astronauts in the International Space Station

**DOI:** 10.1371/journal.pone.0156190

**Published:** 2016-05-18

**Authors:** Masahiro Terada, Masaya Seki, Rika Takahashi, Shin Yamada, Akira Higashibata, Hideyuki J. Majima, Masamichi Sudoh, Chiaki Mukai, Noriaki Ishioka

There is an error in the fifth sentence of the last paragraph of the introduction. The correct sentence is: Recently, analysis of skin from space-flown mice showed that prolonged exposure to space conditions might induce skin atrophy and dysregulate the hair follicle cycle.

There are errors in the last two sentences of the first paragraph of the discussion. The correct sentence is: Neutelings et al. investigated skin from space-flown mice and suggested that prolonged exposure to space environment might induce skin atrophy and deregulate hair follicle cycle, although they reported that the number of hair follicle in anagen increased in the space environment. Considering the FGF18 function on hair cycle, our results are not inconsistent with their findings.

## References

[1] Terada M, Seki M, Takahashi R, Yamada S, Higashibata A, Majima HJ, et al (2016) Effects of a Closed Space Environment on Gene Expression in Hair Follicles of Astronauts in the International Space Station. PLoS ONE 11(3): e0150801 doi: 10.1371/journal.pone.0150801 27029003 10.1371/journal.pone.0150801 PMC4814050

